# Effects of Hydration on Transthyretin Conformational Dynamics and Oligomerization

**DOI:** 10.1021/acs.biochem.5c00589

**Published:** 2025-11-12

**Authors:** Jared Hampton, Carter Lantz, Robert L. Rider, Sangho D. Yun, Arthur Laganowsky, David H. Russell

**Affiliations:** Department of Chemistry, 14736 Texas A&M University, College Station, Texas 77843, United States

## Abstract

Transthyretin (TTR) is a 56 kDa tetrameric protein complex that transports thyroxine and retinol but can misfold, causing amyloid diseases, such as senile systemic amyloidosis, familial amyloid cardiomyopathy, and familial amyloid polyneuropathy. Previous studies have found that TTR aggregation is initiated when tetramers disassemble into monomers, dimers, and trimers, which misfold and assemble into heterogeneous oligomers. These oligomers are thought to be cytotoxic, yet their formation and composition remain poorly understood. To investigate monomer misfolding, ion mobility-mass spectrometry (IM-MS) was applied to wild-type TTR (wtTTR) and the pathogenic L55P variant under varying pH conditions. IM-MS revealed that acidic pH promotes extended monomer conformations for both wtTTR and L55P. Additionally, L55P showed a higher abundance of extended conformations that are attributed to its increased amyloidogenicity. Orbitrap-based charge detection mass spectrometry is used via the direct mass technology (DMT) mode to evaluate oligomeric species, revealing that acidic pH and lower temperatures promote oligomerization and L55P formed oligomers more readily than wtTTR. Together, these results show that oligomerization and conformational changes depend on solution pH, temperature, and proteoform, supporting the role that changes in hydration play in TTR aggregation. More broadly, these findings demonstrate the complementary strengths of IM-MS and DMT for characterizing aggregation intermediates and provide new insights into TTR aggregation.

## Graphical abstract

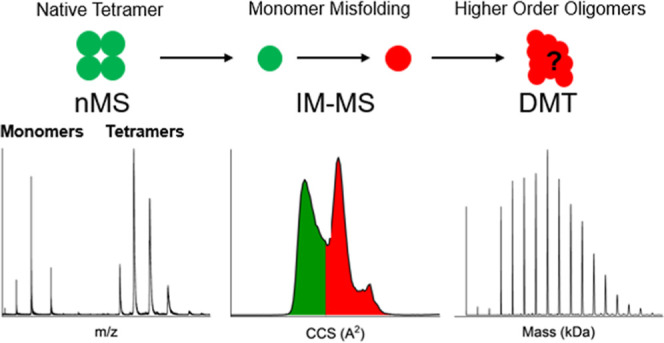

## Introduction

Transthyretin (TTR) is a tetrameric protein complex that transports thyroxine and retinol in cerebral spinal fluid and plasma.^1^ Wild-type TTR (wtTTR) is intrinsically prone to amyloid formation and is linked to senile systemic amyloidosis, a late-onset amyloidosis that primarily affects individuals over the age of 80.^2–5^ In addition, more than 100 naturally occurring TTR mutations have been identified, with several pathogenic variants linked to hereditary amyloid diseases such as cardiomyopathy and polyneuropathy.^6–8^

Previous studies of TTR amyloidosis have primarily focused on fiber formation, using analytical techniques such as thioflavin T (ThT) fluorescence assays, nuclear magnetic resonance spectroscopy, and cryogenic electron microscopy.^9–12^ The dynamics of tetramer stability have also been explored through subunit exchange (SUE) experiments, which examine the assembly and stability of hybrid tetramers formed in individuals expressing both wild type and mutant TTR proteoforms.^13,14^ These studies typically employ structural tags, chromatographic methods, and mass spectrometry to monitor SUE.^15–17^ In addition, TTR misfolding has been investigated with circular dichroism spectroscopy and ion mobility-mass spectrometry (IM-MS).^18,19^ IM-MS has also been applied to investigate the oligomerization of other amyloidogenic proteins, including amyloid-β and α-synuclein, where changes to conformer compaction and extension have been linked to misfolding and aggregation as well as assigning oligomeric state.^20–22^ Despite these advances, resolving intermediate higher-order oligomers remains challenging owing to their heterogeneity, which makes them difficult to characterize with conventional biophysical techniques. Understanding the formation and structural features of these oligomeric species is critical, as they are increasingly suspected to be the cytotoxic species rather than the amyloid fibrils.^23^

Charge detection mass spectrometry (CDMS) is an emerging technique that enables the analysis of highly heterogeneous and high mass samples by directly determining ion masses through simultaneous measurement of *m*/*z* and charge for individual ions.^24^ Unlike conventional native mass spectrometry, which requires well-resolved charge state or isotopic distributions to calculate molecular weight, CDMS bypasses this limitation and is available on custom-built electrostatic linear ion traps and commercial Orbitrap mass analyzers.^25–27^ CDMS has been applied to characterize high mass analytes such as recombinant adeno-associated viruses and heterogeneous systems such as virus spike proteins.^25,28–30^ In addition, CDMS has been used to study protein oligomerization, including antibodies and bovine serum albumin oligomers.^31–33^ More recently, CDMS has been integrated with complementary techniques such as IM-MS, electron capture dissociation, and molecular dynamics simulations, as demonstrated by Xi et al. and Kuo et al. in studies of peptide self-assembly, including liraglutide, a glucagon like peptide-1 (GLP-1) analog.^34,35^

Here, IM-MS and orbitrap-based CDMS using direct mass technology (DMT) mode were used to investigate the formation of soluble higher-order TTR oligomers and the role of hydration in their assembly. IM-MS revealed that lowering pH increases the abundance of extended monomer conformations, interpreted as misfolded species, which are more prominent in the pathogenic L55P variant, consistent with prior IM-MS studies of TTR.^19,36^ DMT further showed that acidic conditions promote oligomerization owing to the free energy landscape shifting toward misfolded states.^37^ Incubation temperature studies were performed at pH 4.4, the optimum pH for TTR fiber formation.^38^ Under these conditions, colder temperatures promoted oligomerization, whereas warmer temperatures delayed it, supporting the influence of hydration in stabilizing the native tetramer as suggested by recent SUE studies by Lantz et al.^39^ While this previous work implicated water molecules and hydration networks in the formation of hybrid TTR tetramers, the effect of hydration on TTR oligomerization has not been directly examined. Across the conditions tested, L55P consistently formed oligomers more readily than wtTTR. Together, these findings demonstrate the complementary utility of IM-MS and DMT for resolving amyloidogenic intermediates and demonstrate how hydration, altered by pH, temperature, and mutation, influences TTR aggregation.

## Experimental Section

### TTR Expression and Sample Preparation

TTR [Accession ID: P02766 (UniProtKB/Swiss-Prot)] was expressed following a previously described protocol.^39^ TTR was buffer-exchanged into 100 mM ammonium acetate (Millipore, Burlington, MA) at the desired pH using Bio-Spin 6 SEC columns (BioRad, Hercules, CA). The ammonium acetate buffer was prepared with LC–MS grade water (Millipore), and its pH was adjusted by using Optima LC–MS grade acetic acid (Fisher Scientific, Pittsburgh, PA). The final concentration of TTR was adjusted to 42.5 μM (monomer) for incubation for up to 24 h. For temperature-controlled conditions, samples were incubated at 37 °C using a MyTemp Digital Incubator (Benchmark Scientific, Sayreville, NJ) and at 4 °C in a cold room. Samples were analyzed using gold-coated pulled borosilicate capillaries prepared with a P-1000 micropipette puller (Sutter Instrument, Novato, CA) and a Leica EM ACE200 sputter coater (Leica Microsystems, Wetzlar, Germany).

### Orbitrap Charge Detection Mass Spectrometry/Direct Mass Technology Measurement

All measurements were performed on an ultrahigh mass range mass spectrometer (ThermoFisher Scientific, Bremen, Germany) equipped with the DMT software upgrade to perform Orbitrap-based CDMS following a previously described procedure.^40^ Briefly, ions were lowered to single ion level abundance, and then the DMT acquisition mode was enabled, allowing for the simultaneous measurement of *m*/*z* and charge of ions. DMT measurements were acquired using an HCD cell trapping gas pressure of 0.3–0.5, at 200,000 resolution (at 400 *m*/*z*), collecting until 10,000 ions were accepted, typically taking between 20 and 45 min. Instrument parameters included a 50 eV in-source CID energy, a 50 V HCD energy, a −10 V in-source trapping voltage, and a spray voltage of 1.1 to 1.6 kV. Measurements were taken every 3 h over the first 12 h and again at 24 h. DMT data were processed using the STORIboard software from Proteinaceous (Chicago, IL) with the “voting v3” charge assignment method.^41^ The exact parameters used for processing can be found in the Supporting Information (Table S1). Data analysis and visualization were performed using OriginPro (OriginLab, Northampton, MA) and custom Python scripts.

### Ion-Mobility Mass Spectrometry

IM-MS measurements were performed on an Agilent 6560 IM-QTOF mass spectrometer (Agilent Technologies, Santa Clara, CA) by using the same gold-coated borosilicate capillaries prepared for DMT analysis. Each IM-MS acquisition was collected over a 3 min period using single-field ion mobility mode. Instrument parameters included a spray voltage of 1600 V, a gas temperature of 250 °C, a drying gas flow rate of 5 L/min, a fragmentor voltage of 300 V, and a collision cell voltage of 50 V. Drift time was converted to collision cross section (CCS) using the single field method as described previously.^42^

## Results

To investigate TTR aggregation, acidic conditions were used to promote the formation of amyloidogenic species. Three pH values, 3.4, 4.4, and 5.4, were selected for this study based on previously observed differences for promoting fibril formation.^43^ Incubation temperature was also varied, given its known influence on tetramer stability from prior aggregation and SUE studies.^39,44^ Both lower and higher temperatures were found to impact oligomerization, likely owing to the role of water dynamics in stabilizing the tetramer, as supported by earlier findings.^45^ To assess the impact of different mutants, L55P was incubated under identical conditions as wtTTR. L55P was selected owing to it being known as the most pathogenic mutant of TTR.^46^ This study investigated soluble higher-order oligomers in solution, excluding insoluble aggregates.

### IM-MS Detects pH-Dependent TTR Monomer Misfolding

Figure 1 shows the native mass spectra of wtTTR (Figure 1a–c) and L55P (Figure 1d–f) at solution pH values of 3.4, 4.4, and 5.4. For both wtTTR and L55P, more acidic conditions yield a higher relative abundance of monomeric species in the mass spectrum compared to higher pH values. The increased tetramer abundance at pH 5.4 reflects the higher stability of the native tetramer, whereas acidic conditions (pH 3.4 and pH 4.4) promote tetramer dissociation and initiate TTR misfolding. The observation of primarily monomer and tetramer signals suggests that the monomer is the key aggregation precursor, consistent with the downhill polymerization mechanism.^43^ This behavior parallels SDS-PAGE results, where more acidic conditions reduced tetramer abundance for the L55P variant.^46^

**Figure 1:**
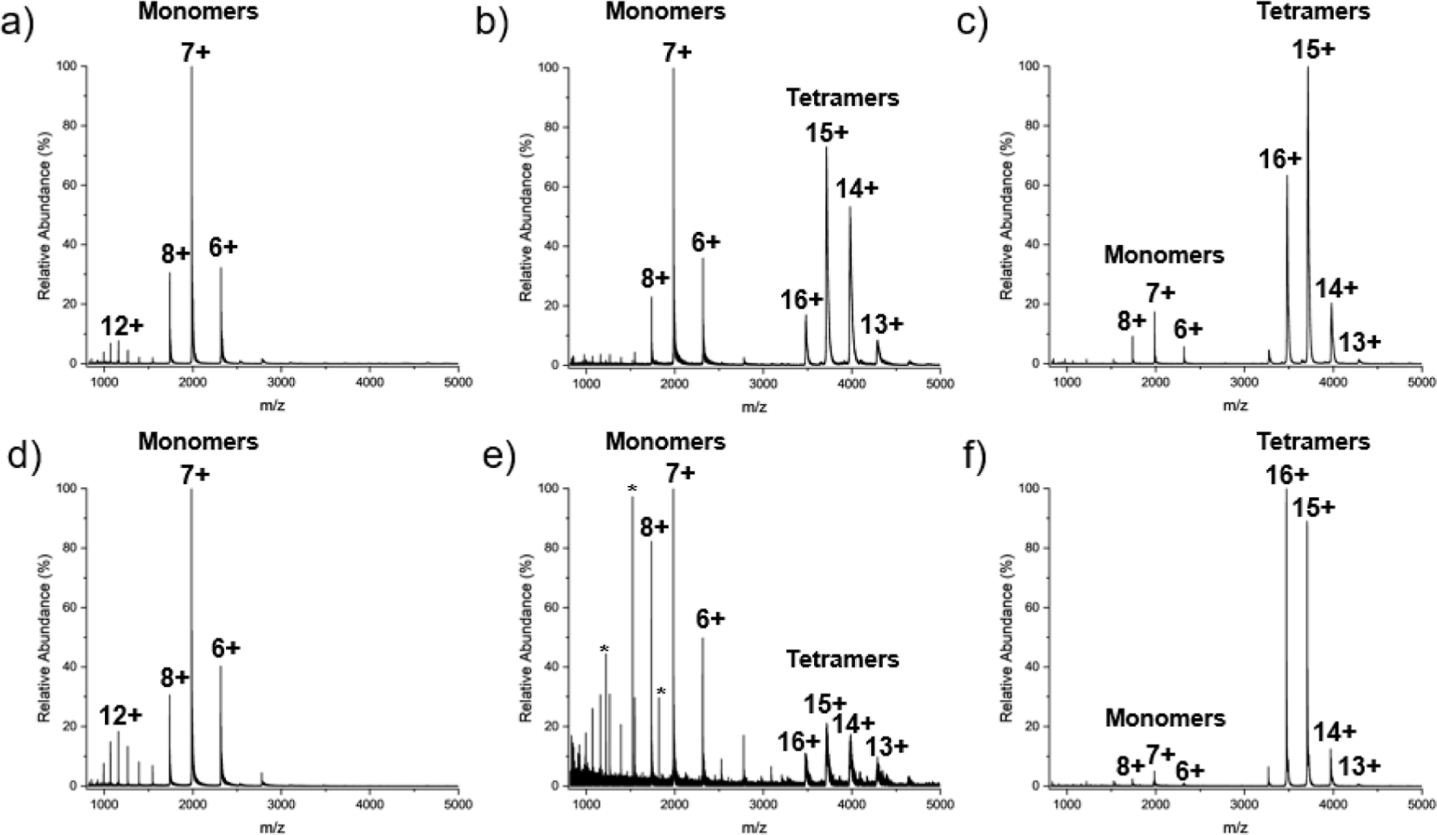
Mass spectra acquired on an Agilent 6560 IM-MS Q-TOF mass spectrometer. wtTTR (a–c) and L55P (d–f) were analyzed after a 24 h incubation at RT in 100 mM AmAc at pH 3.4 (a,d), pH 4.4 (b,e), and pH 5.4 (c,f). Peaks marked with * indicate residual tune mix ions present in the spectra.

IM-MS was employed to examine these misfolding events by monitoring conformational changes under different pH conditions. Results from IM-MS of the 8+ monomer for wtTTR and L55P are shown in Figure 2. For wtTTR (Figure 2a), progressively more extended conformations were observed as the pH decreased, with pH 3.4 showing the greatest abundance of extended conformers compared with pH 4.4 and 5.4. At pH 3.4, the compact conformer also exhibited a higher CCS (1706 Å^2^) relative to those at pH 4.4 (1687 Å^2^) and pH 5.4 (1689 Å^2^), indicating more extended states under more acidic conditions. Deconvoluting the mobiligrams reveals higher conformer abundances at extended conformations for more acidic pH. More information on CCS, peak width, and the full width at half-maximum (fwhm) for wtTTR can be found in the Supporting Information (Table S2).

**Figure 2:**
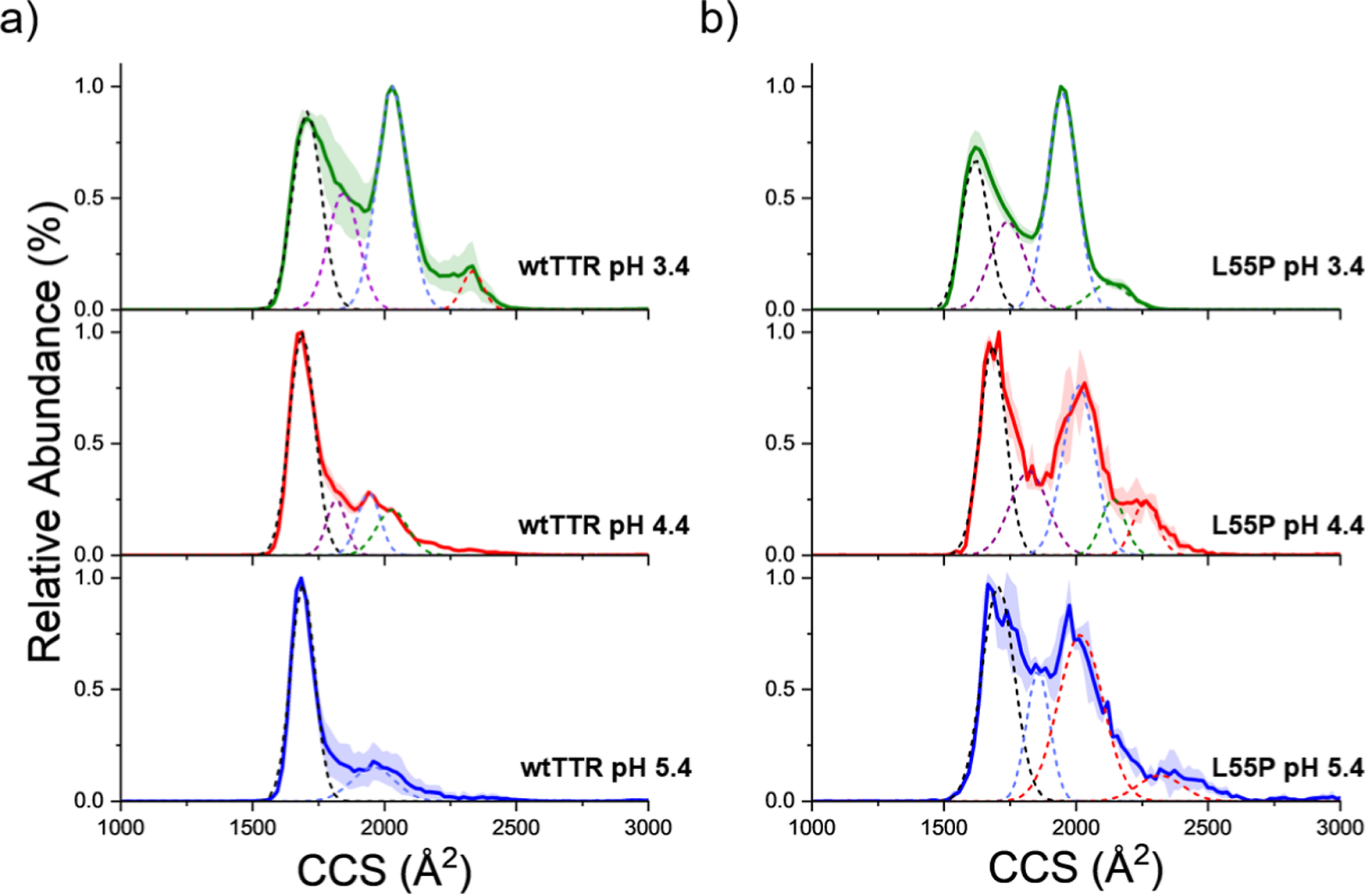
IM-MS of the 8+ monomer for (a) wtTTR and (b) L55P after 24 h of incubation at RT in 100 mM AmAc at pH 3.4 (green), 4.4 (red), and 5.4 (blue). Shaded regions represent standard deviation of replicates (*n* = 3). Dashed lines indicate fits corresponding to distinct monomer conformations.

For L55P (Figure 2b), the conformers observed at pH 3.4 are like wtTTR but exhibited lower CCS values across the 4 conformations (Table S3). At pH 4.4 and 5.4, however, L55P has a greater abundance of extended conformations relative to wtTTR. The increased prevalence of these extended states may explain why L55P aggregates more rapidly than wtTTR. The other monomer charge states and tetramer conformations for wtTTR and L55P are provided in Figures S1–S3. Detailed values of L55P for the CCS, peak areas, and fwhm are provided in Table S3. These findings are consistent with solution-based analytical methods such as analytical ultracentrifugation and ANS binding assays, which also show increased monomer misfolding under more acidic conditions.^46,47^

Figure 2 reveals a greater abundance of extended conformers for L55P compared to wtTTR, as well as increased unfolding in response to more acidic pH. The same amount of conformers is seen at pH 3.4, indicating a similar response for wtTTR and L55P under those conditions. The increase in conformers and increase in abundance of extended conformers for L55P likely represent distinct unfolding intermediates involved in the aggregation process. Furthermore, the broader fwhm of L55P conformers relative to wtTTR suggests greater structural heterogeneity within these populations. Similar behavior was observed for the monomeric mutant F87A (Figure S4), where more acidic conditions promoted more extended conformations, consistent with the trends observed for wtTTR and L55P. To rule out instrument-related effects, the Fragmentor voltage was lowered (Figure S5), which produced no observable change in conformer distribution.^19^

### DMT Resolves Heterogeneous TTR Oligomers

A comparison of a traditional ensemble native mass spectrum with a DMT spectrum resolving heterogeneous higher-order oligomers is shown in Figure 3. In the native mass spectrum (Figure 3a), species above 5000 *m*/*z* cannot be resolved because of the absence of distinguishable charge states or isotopic distributions. In contrast, the DMT spectrum (Figure 3b) enables the assignment of mass and thus the oligomeric state for these species. Corresponding 2D heatmaps of charge vs *m*/*z* and charge vs mass (Figure S6 in the Supporting Information) show that with the additional measurement of charge, the species observed can be separated from one another when overlapping at the same *m*/*z* region. Additionally, as the mass is increased, a linear increase in charge can be observed (Figure S6b). The measured masses are within 1% of the theoretical values for each oligomer (Table S3), with excess mass likely arising from common adducted species (Na^+^, K^+^, Zn^+^).^48^

**Figure 3:**
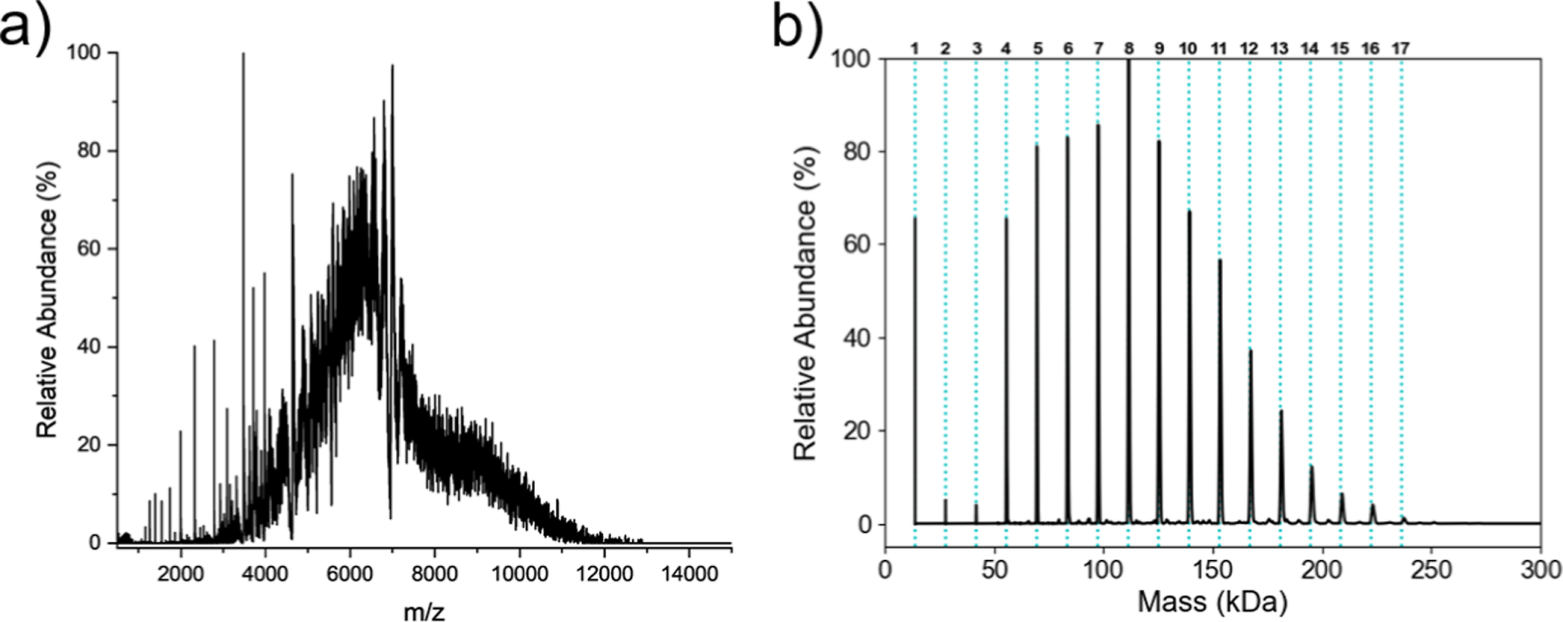
Comparison of (a) the native mass spectrum and (b) the DMT spectrum of TTR in 100 mM AmAc at pH 3.4 after 1 day of incubation at 4 °C. Bold numbers above panel (b) indicate the number of TTR subunits corresponding to the theoretical masses marked by the blue dashed lines.

### The Effect of pH and Incubation Temperature on TTR Oligomerization

The effect of solution pH on TTR oligomerization was examined at pH 3.4, 4.4, and 5.4 over a 24 h incubation period at room temperature (Figure 4). At pH 3.4 (Figure 4a), oligomers are present at the initial time point and remained consistent throughout the time course, including species containing up to 19 subunits. The most dynamic behavior occurred at pH 4.4 (Figure 4b), where only the monomer, tetramer, and octamer were initially present. After 9 h of incubation, signals for dimers, trimers, and pentamers emerged (Figure 4b), indicating the disassembly of the tetramer. By 24 h, oligomers containing up to 14 monomers were detected. In comparison, at pH 5.4 (Figure 4c), only monomers, tetramers, and octamers were detected across all time points, with no significant change observed. The presence of octamers at both pH 4.4 and 5.4 is consistent with previous IM-MS experiments by Ruotolo et al.^19^ Based on these findings, pH 4.4 was selected for subsequent incubation temperature studies, as it showed the greatest changes in oligomerization over time and has previously been identified as the optimal pH for TTR aggregation.^12,38^

**Figure 4:**
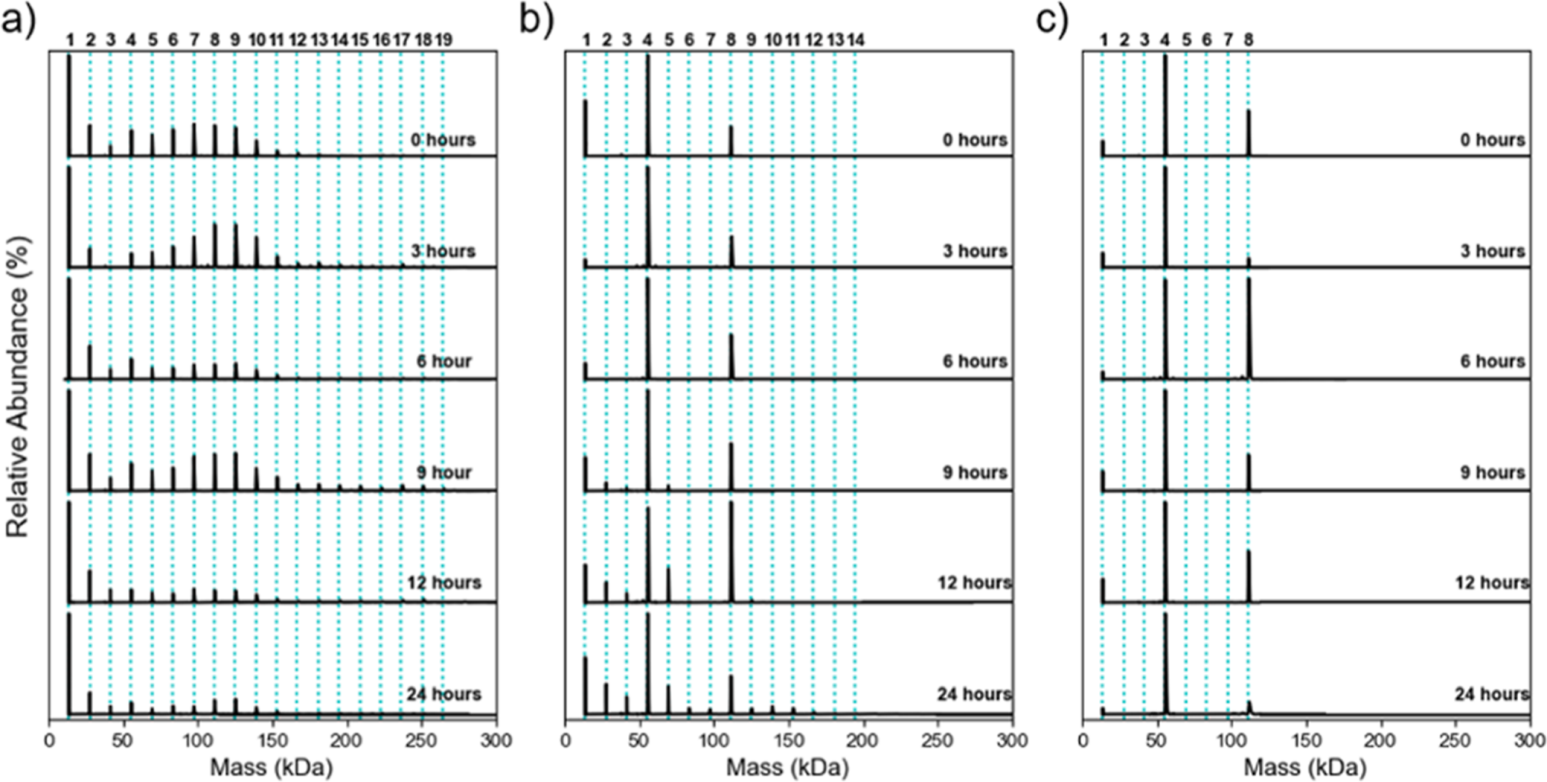
DMT spectra of TTR oligomers in 100 mM AmAc at 21 °C at solution pH values of (a) 3.4, (b) 4.4, and (c) 5.4 over a 24 h period. Bold numbers above each graph indicate the number of TTR subunits, while blue dashed lines mark the theoretical mass of the corresponding oligomers. Bolded time points indicate the incubation time at which each spectrum was recorded.

The effect of incubation temperature on the TTR oligomerization was investigated at pH 4.4 for both wtTTR and the L55P mutant as shown in Figure 5 (wtTTR: a–c; L55P: d–f). For wtTTR, incubation at 4 °C (Figure 5a) led to the appearance of oligomers containing up to 8 subunits after 6 h, appearing 3 h earlier than that observed at 21 °C (Figure 5b). In contrast, incubation at 37 °C inhibited oligomer formation, with only monomer, tetramer, and octamer species present. For L55P, oligomerization was promoted, with 4 and 21 °C oligomers formed at 3 h (Figure 5d,e). Figure S7 shows oligomer formation at 4 and 21 °C after 1 h of incubation. At 37 °C, oligomer formation was delayed until after 6 h, although dimer and trimers were visible by 3 h (Figure 5f), consistent with tetramer disassembly.^13,15^ Overall, these results indicate that colder temperatures promote oligomerization, whereas warmer conditions inhibit it.

**Figure 5:**
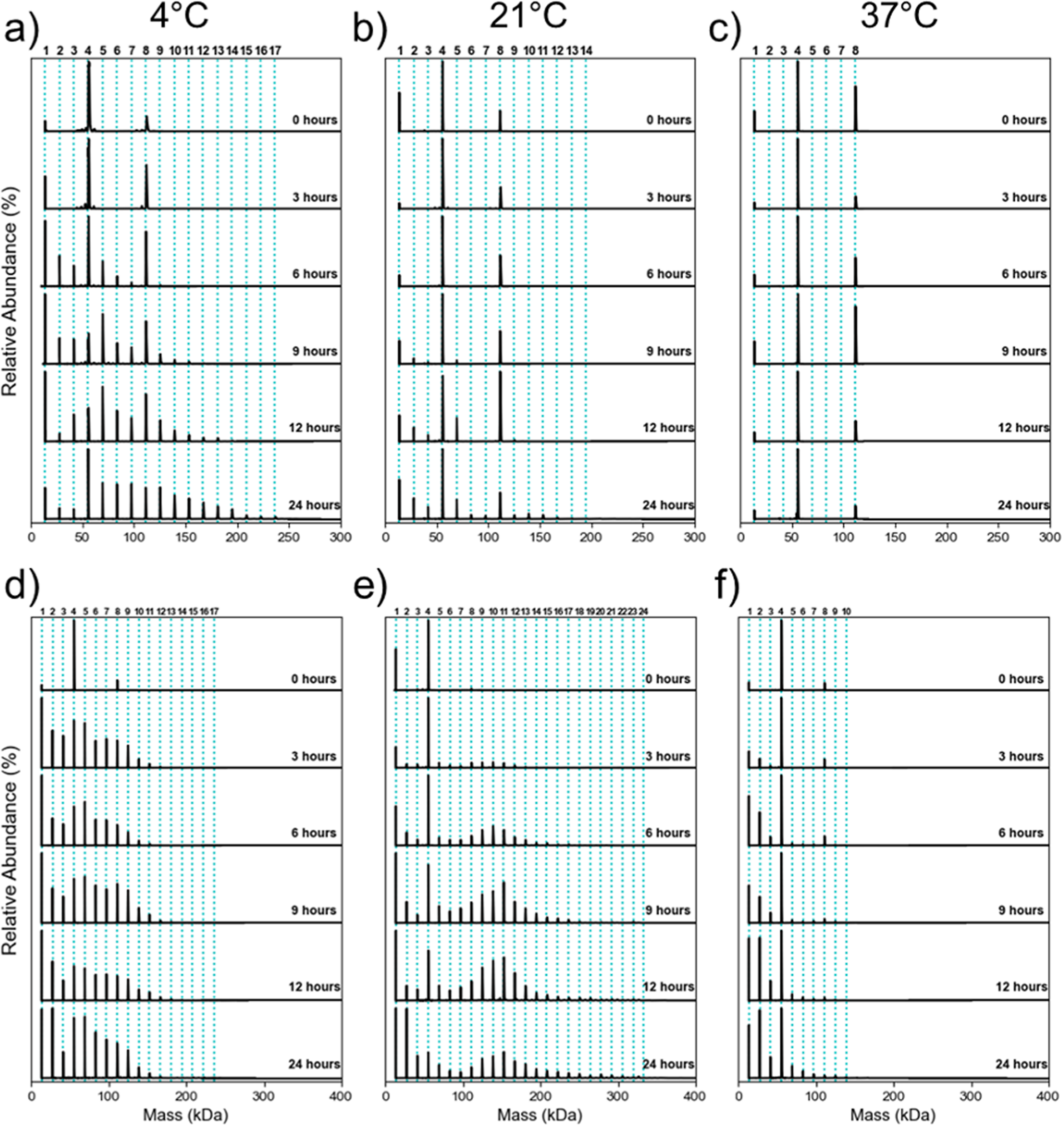
Effect of incubation temperature over 24 h for wtTTR (a–c) and L55P (d–f) at pH 4.4 in 100 mM AmAc. Incubation temperatures are 4 °C (a,d), 21 °C (b,e) and 37 °C (c,f). Bold numbers above each graph indicate the number of TTR subunits, while blue dashed lines mark the theoretical masses of the corresponding oligomers. Times displayed to the right of each DMT spectrum indicate the incubation time at which the spectrum was recorded. Figure 5b is identical to Figure 4b and is included here for easier visual comparison across temperatures.

Comparing the oligomerization of wtTTR and L55P reveals several differences across pH and temperature conditions. L55P response to pH 3.4 and 5.4 can be found in Figure S8. Similar behavior to wtTTR (Figure 4a) is observed, where higher-order oligomer formation is observed right away at pH 3.4 (Figure S8a) but is not observed at pH 5.4 (Figure S8b). After 24 h at 4 °C, wtTTR oligomers contained up to 17 subunits, whereas L55P contained up to 19 subunits. At 21 °C, wtTTR produced oligomers of up to 14 subunits, while L55P formed larger species containing as many as 24 subunit oligomers, whose masses can be found in Table S4. At 37 °C, wtTTR showed no oligomerization beyond the octamer, whereas L55P forms up to 10 subunit oligomers. These results demonstrate that L55P undergoes more extensive oligomerization than wtTTR under all incubation temperatures tested. It should be noted that the oligomers detected here are smaller than those expected by microscopy during fibril formation, likely owing to the fact that larger aggregates (larger oligomers and protofibrils) are insoluble in solution.^49^ Instrument parameters were varied to assess their effect on oligomer transmission (Figure S9), and the current parameters were found to be optimal for analysis.

## Discussion

Differences in solution conditions affected both the onset and composition of higher-order TTR oligomers. Acidic conditions promoted oligomerization, consistent with previous studies that acidic conditions shift the free energy landscape to favor misfolded, aggregation-prone states over the native tetrameric form.^37^ This trend is evident in Figure 1 where the tetramer abundance decreases with increasing acidity. Oligomer formation at pH 3.4 and 4.4 (Figure 4) may be linked to TTR’s isoelectric point (pI) of 5.4, as conditions near or below the pI enhance hydrophobic interactions and promote aggregation.^50^ In contrast, pH 5.4 is above the pI and, therefore, lacks the hydrophobic interactions necessary for oligomer formation. This is observed in Figure 2 where higher abundance of extended monomer conformations under acidic conditions suggests exposure of hydrophobic regions, altered hydration, and the initiation of oligomerization. Prior studies in phosphate and KCl buffers have identified pH 4.4 as the optimal for fiber formation; the oligomer formation observed in Figure 4 suggests that this may not hold true for oligomerization.^47^ However, ammonium acetate has been found to increase protein complex stability and promote compact conformations compared to traditional biological buffers.^51^

IM-MS was used to monitor misfolded monomers, which can be seen with the 8+ charge-state monomer (Figure 2). At pH 3.4, wtTTR and L55P show nearly identical 8+ mobiligrams, and both display highly charged monomers indicative of more extended conformations (Figure 1a,d). At pH 4.4 and 5.4, however, L55P has a greater abundance of extended conformers than wtTTR. This greater abundance of extended conformers could explain the L55P propensity for aggregation, as the extended state is likely more prone to misfolding and oligomer formation than compact conformations. Notably, at pH 5.4, where aggregation is not observed for either mutant, L55P still has a high abundance of extended conformers (Figure 2b), which could explain its inherent propensity for aggregation. These additional conformers could be additional unfolded species that contribute to oligomer formation. The increased abundance of extended conformations in L55P could arise from a trans-to-cis isomerization associated with the leucine-to-proline substitution, which likely increases the CCS and reflects monomer misfolding.^52^ Additionally, perturbation of the β sheet region caused by the L55P mutation may further contribute to the extended conformations observed.^53^ Finally, the increase in monomer abundance with decreasing pH underscores that acidic conditions promote tetramer disassembly (Figure 1), thereby overcoming the rate-limiting step of TTR amyloidosis and initiating aggregation.^43^

The increased abundance of extended monomer conformations under more acidic conditions supports the downhill polymerization mechanism of TTR, in which monomers misfold and subsequently assemble into aggregates.^54^ To examine the effect of incubation temperature and protein hydration on TTR aggregation, cold (4 °C) and warm (37 °C) conditions were compared (Figure 5). Because TTR aggregation is limited by the rate of tetramer dissociation, colder temperatures promoted a higher degree of oligomer formation for wtTTR (Figure 5a), consistent with previous studies on TTR fiber formation.^12^ In contrast, at 37 °C, oligomer formation was not observed (Figure 5c). Banerjee et al. attributed this temperature dependence to conserved water molecules that stabilize the tetramer, classified as “hot” and “cold” based on whether their exchange with bulk water is fast or slow.^45,55^ In earlier SUE studies, between mutant and wtTTR, disruption of these water networks at lower temperatures promotes tetramer destabilization and SUE process.^39^ Additional support comes from high-pressure studies of TTR, showing that pressure-induced conformational changes increase water susceptibility, destabilizing the tetramer and promoting aggregation through altered hydration states.^56^ Because tetramer disassembly is the rate-limiting step in TTR aggregation, perturbations that weaken water-mediated stabilization, such as cold temperatures, increase hydration within hydrophobic regions (the dimer interface formed by contacts between the AB and GH loops), thereby promoting tetramer disassembly owing to disrupted hydration states.^57,58^

L55P promoted oligomer formation more readily than wtTTR at all incubation temperatures, consistent with its known amyloidogenicity.^46^ One possible explanation is the increased solvent exposure in L55P, supported by variable-temperature studies showing higher average charge state relative to wtTTR.^59^ Interestingly, oligomers formed by L55P at 21 °C (Figure 5e) and 37 °C (Figure 5f) contained more individual subunits than those formed by wtTTR (Figure 5b,c), an unexpected finding given that TTR fibers across proteoforms are structurally similar.^11^ Differences in hydration may contribute to this effect, as hydrogen–deuterium exchange (HDX) studies have shown increased exchange in residues 30–40 for L55P compared to wtTTR, indicating greater solvent accessibility.^60^ Another possible explanation for the observed temperature dependence is that higher temperatures promote more efficient aggregate formation, thereby depleting the oligomer formation.^46^ This underscores the importance of studying oligomer formation across different TTR mutants, as illustrated by prior variable-temperature studies of the pathogenic V30M variant, which also showed altered charge states.^59,61^ Future DMT studies may help clarify such differences in aggregation mechanisms that remain unresolved in past studies of TTR.^12^

Across the DMT spectra collected for wtTTR at different incubation temperatures and pH values, octamer signals can be observed when no other higher-order oligomer species are formed (Figures 4 and 5a–c). Similar species were previously reported by Ruotolo et al. in IM-MS experiments.^19^ These species are present for wtTTR even when other oligomer species do not form, which suggests that it could be a native species rather than a precursor species for aggregation as shown for wtTTR at pH 4.4 prior to 9 h of incubation (Figure 5b) and pH 5.4 (Figure 5c). By contrast, the L55P variant (Figure 5d–f) forms octamers less readily than wtTTR, which is attributed to its reduced tetramer stability compared to wtTTR.^15^

## Conclusions

In summary, IM-MS and Orbitrap-based CDMS via DMT were applied to investigate how hydration influences TTR aggregation. IM-MS showed distinct conformations for the TTR 8+ monomer with more extended conformations observed at lower pH and for the L55P mutant, suggesting an increased propensity for oligomerization under these conditions. Using DMT further showed that oligomerization depends on solution pH, incubation temperature, and TTR proteoform. Acidic conditions promoted oligomer formation by shifting the free energy landscape to favor tetramer disassembly and monomer misfolding, while colder incubation temperatures enhanced oligomerization through changes in hydration, destabilizing the tetramer. Compared to wtTTR, the L55P mutant formed larger oligomers at earlier time points, consistent with its higher aggregation propensity.

Studies are underway to investigate TTR higher-order oligomer formation, in additional pathogenic mutants, such as V30M and V122I, and under more physiologically relevant conditions (i.e., pH 7.4).^44,62,63^ Further work will also examine the influence of metal ions or drug binding on oligomer formation.^64,65^ Exploring the effect of solvent interactions with heavy water similar to Yee et al. could provide further studies for TTR.^66^ Beyond TTR, this approach may be applied to other amyloid proteins, such as α-synuclein or amyloid-β, to gain insights into their aggregation mechanism. This study establishes a framework for applying IM-MS and CDMS methods to resolve each protomer that other biophysical techniques cannot assess.

## Supplementary Material



## Notes

Methods of TTR expression, DMT, and IM-MS measurements; additional DMT heatmaps; tables of masses of TTR oligomers; STORIboard processing parameters; additional DMT spectrum; IM-MS mass spectra; and mobiligrams from other charge states (PDF)
