# Supplementary material for: Effects of Hydration on Transthyretin Conformational Dynamics and Oligomerization
Source: Biochemistry. 2025 Nov 12;64(23):4571–80. doi: 10.1021/acs.biochem.5c00589 (PMC12676739; doi:10.1021/acs.biochem.5c00589)
Supplement: Supplementary file 1 [file bi5c00589_si_001.pdf]

Supporting Information

Effects of Hydration on Transthyretin Conformational Dynamics and Oligomerization

Jared Hampton, Carter Lantz, Robert L. Rider, Sangho D. Yun, Arthur Laganowsky, David H. Russell*

Department of Chemistry, Texas A&M University, College Station, TX 77843, USA

*Corresponding author; email: russell@chem.edu

Table of Contents | Page

Figure S1	S2
Figure S2	S2
Figure S3	S3
Figure S4	S3
Figure S5	S4
Figure S6	S4
Figure S7	S5
Figure S8	S5
Figure S9	S6
Table S1	S6
Table S2	S7
Table S3	S7
Table S4	S8
Table S5	S8

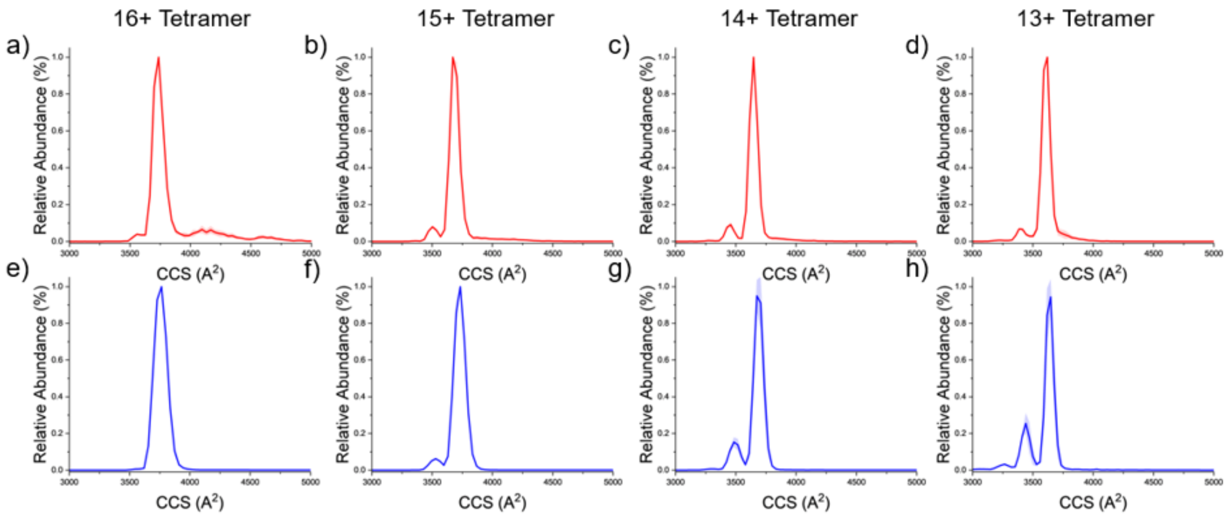

Figure S1: Collision cross sections (CCS) of wtTTR tetramers for the 16+ (a, e), 15+ (b, f), 14+ (c, d), and 13+ (d, h) charge states in 100 mM AmAc at 21 °C after 24-h incubation at pH 4.4 (a-d, red) and pH 5.4 (e-h, blue).

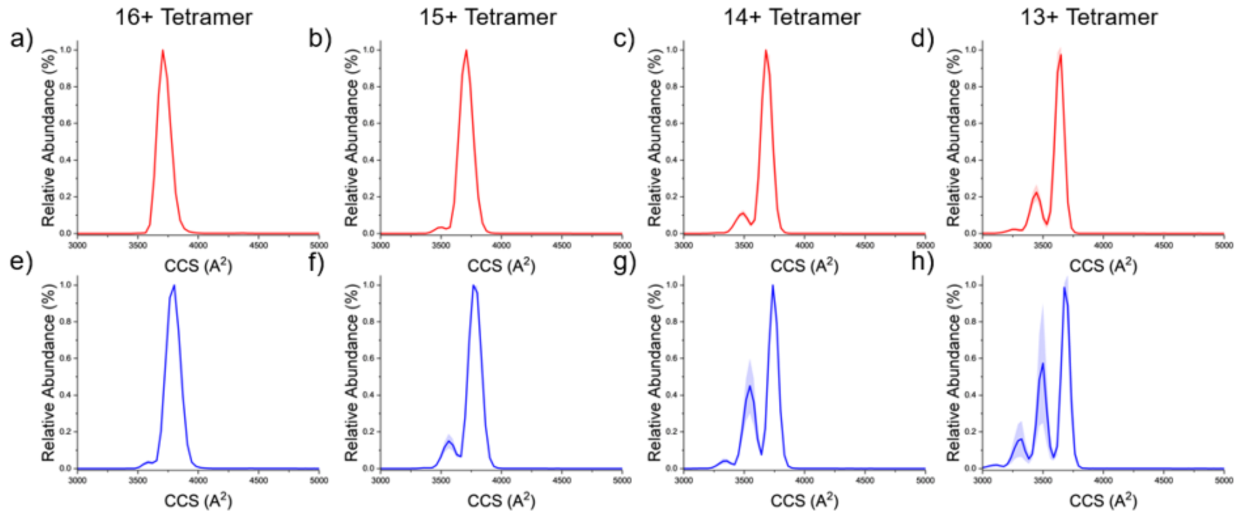

Figure S2: CCS of L55P tetramers for the 16+ (a, e), 15+ (b ,f), 14+ (c ,d), and 13+ (d, h) charge states in 100 mM AmAc at 21 °C after 24-h incubation at pH 4.4 (a-d, red) and pH 5.4 (e-h, blue).

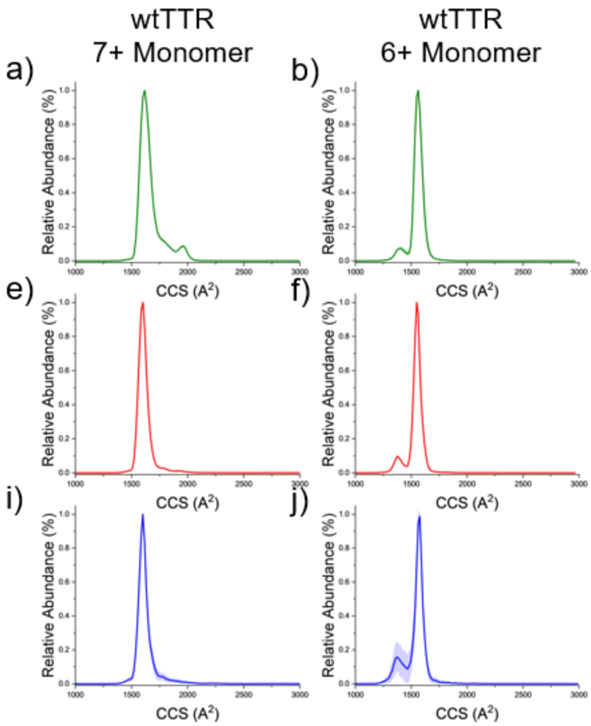

Figure S3: CCS of the 6+ and 7+ charge states for monomers of wtTTR and L55P in 100 mM AmAc incubated at 21 °C for 24 h at pH 3.4 (a-d, green), pH 4.4 (e-h, red), and pH 5.4 (i-l, blue).

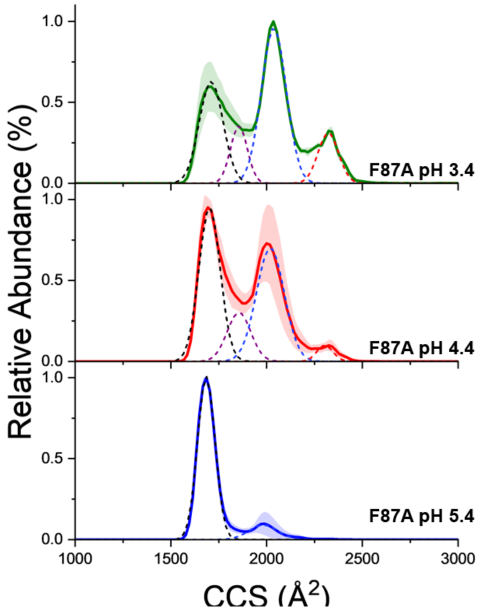

Figure S4: CCS of the 8+ monomer of F87A after 24 h of incubation at RT in 100 mM AmAc at pH 3.4 (green), 4.4 (red), and 5.4 (blue). Dashed lines indicate fits corresponding to distinct monomer conformations.

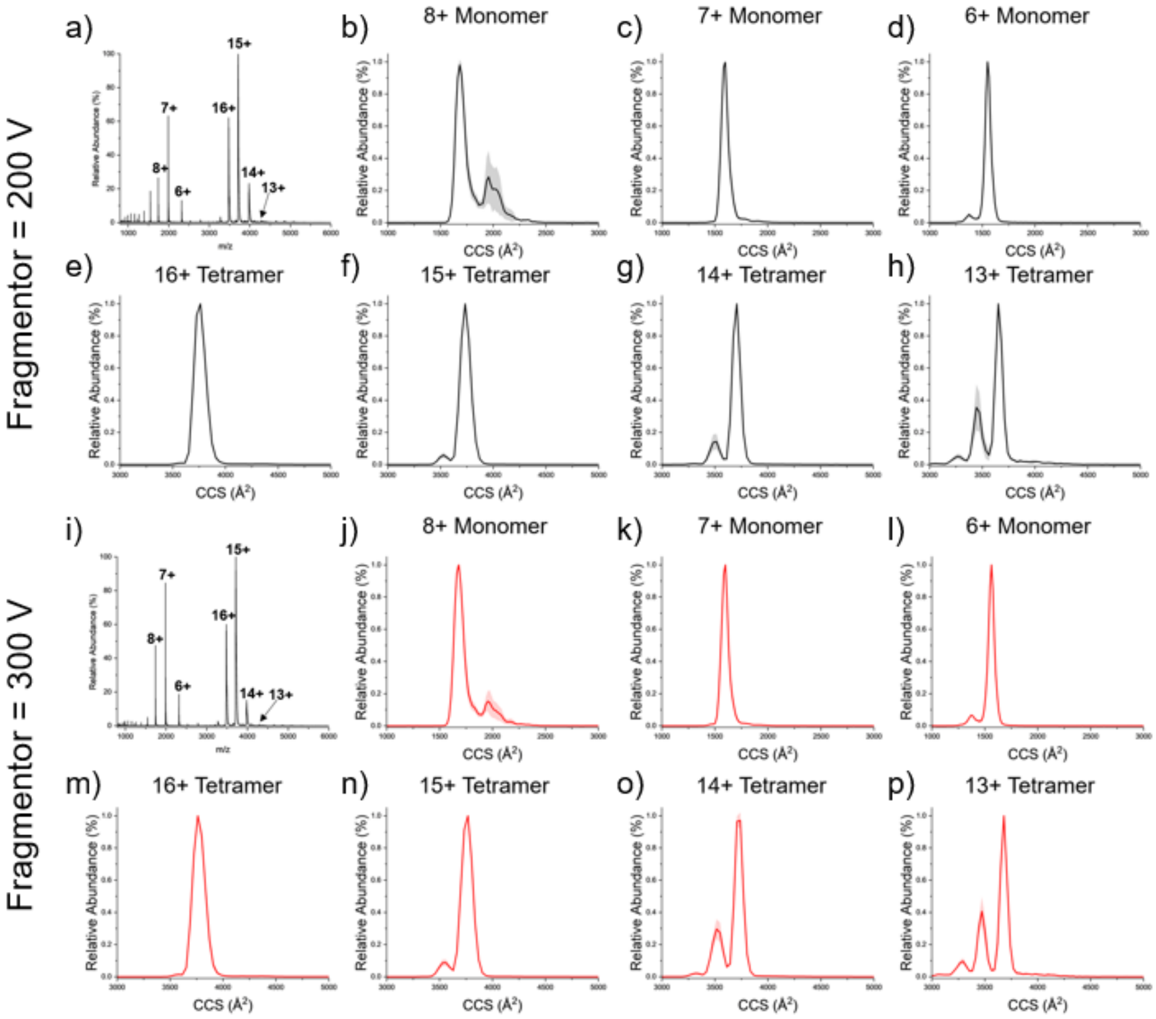

Figure S5: CCS of wtTTR in pH 4.4 100 mM AmAc after 24 h incubation at RT, measured at Fragmentor voltages of 200 V (a-h, black) and 300 V (i-p, red). Shown are the corresponding mass spectra (a, i); CCS of 8+ (b, j), 7+ (c, k), and 6+ (d, l) monomer charge states; and tetramer CCS of 16+ (e, m), 15+ (f, n), 14+ (g, o), and 13+ (h, p) charge states.

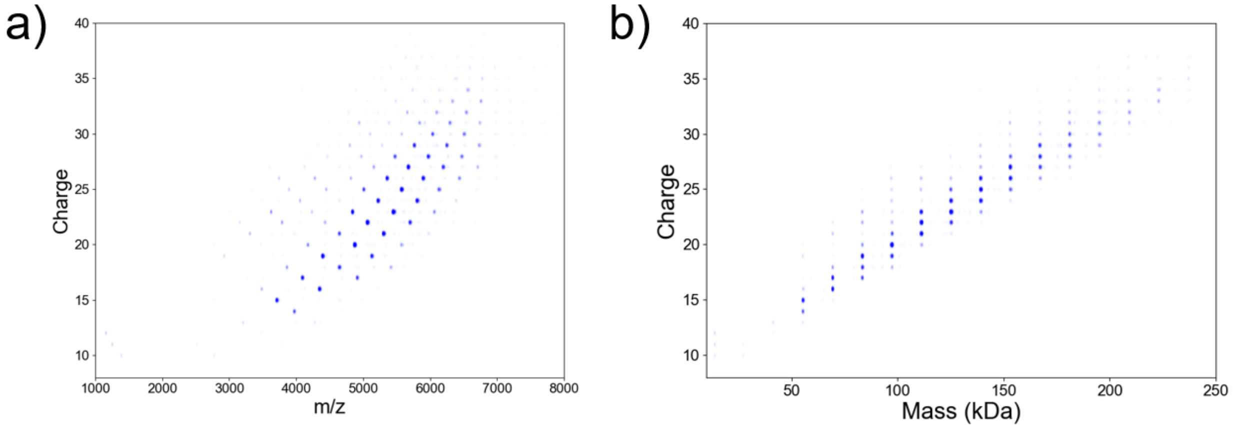

Figure S6: 2D heatmaps of 100 mM AmAc at pH 3.4 and 4 °C (from Figure 1 in main text) showing a) charge vs. m/z and b) charge vs. mass.

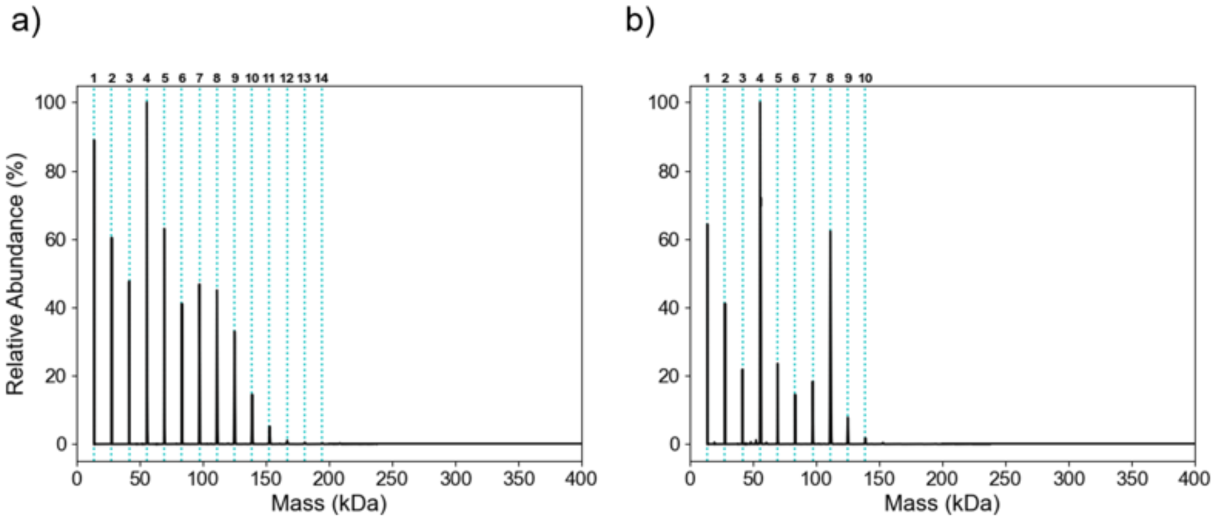

Figure S7: DMT spectra of L55P in 100 mM AmAc at pH 4.4 after 1 h of incubation at a) 4 °C and b) 21 °C. Numbers above each graph indicate the corresponding number of subunits, while blue dashed lines mark the theoretical mass.

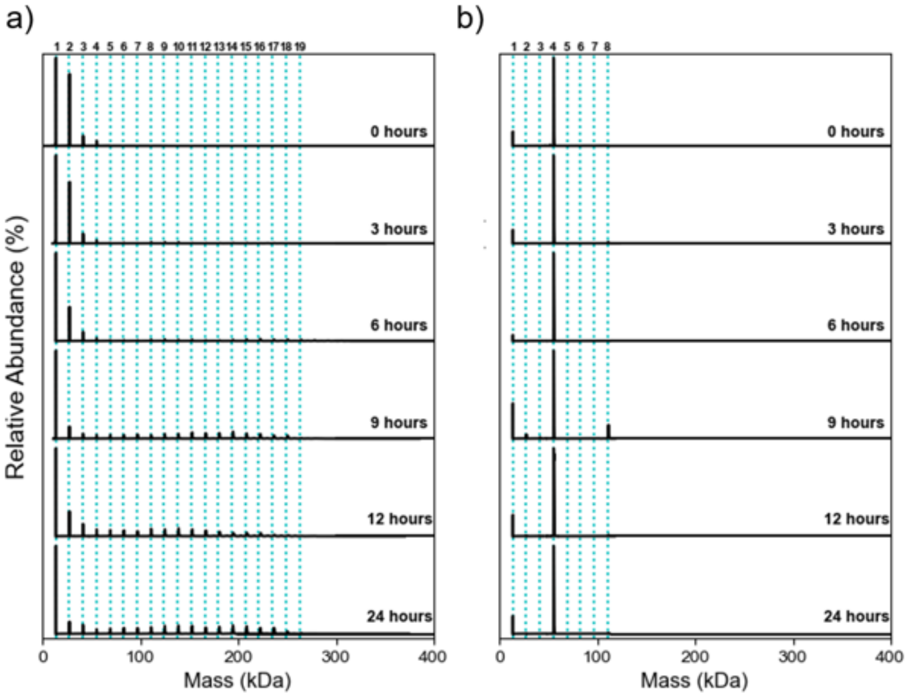

Figure S8: L55P incubated at 21°C in 100 mM AmAc for 24 h. Solution pH values were a) pH 3.4 and b) pH 5.4. Dashed blue lines and numbers at the top indicate the theoretical mass and corresponding number of subunits, respectively. Measurements were taken at the incubation times indicated.

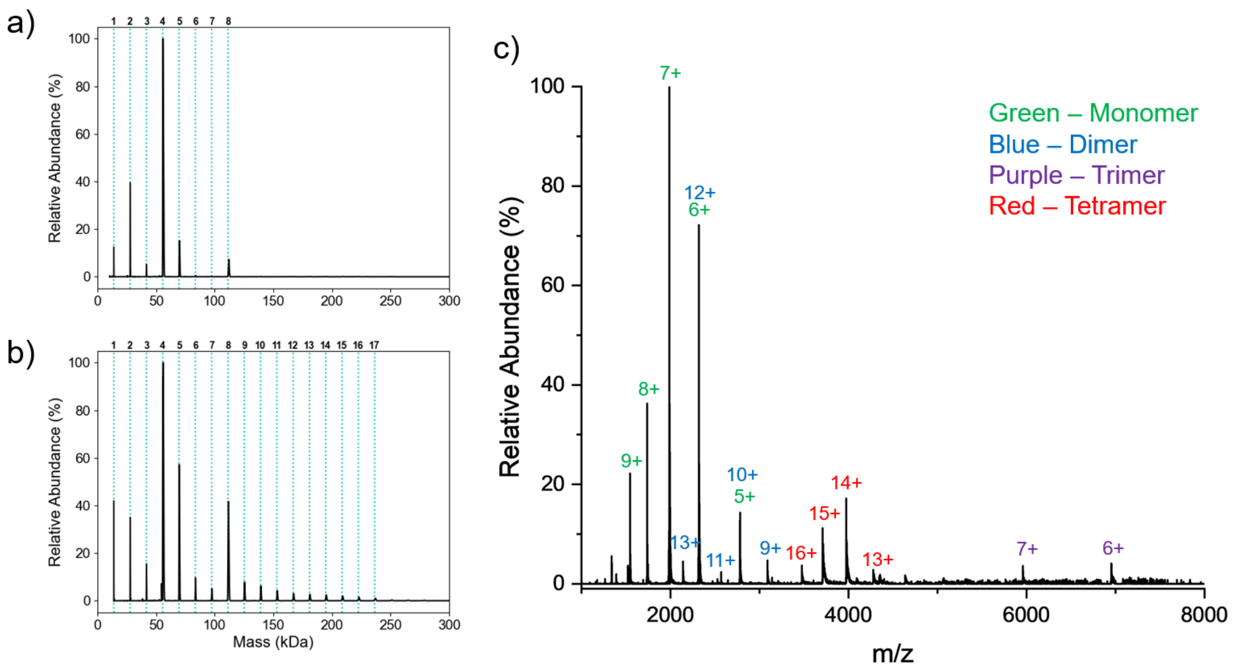

Figure S9: Effect of HCD Cell collision energy (CE) on wtTTR oligomers in 100 mM AmAc at pH 4.4 after 24 h of incubation at RT. CE values are a) 25, b) 50, and c) 75 are shown. DMT spectra are shown in a) and b), while a traditional ensemble mass spectrum is shown in c) due to the absence of observable oligomers seen at CE=75.

Table S1: STORIboard processing parameters used for processing Direct Mass Technology data.

**STORI Processor**	
r^2 Threshold	0.996
Duration Threshold	0.42
Minimum Time of Death	0.2
Minimum Time of Birth	0.1
Signal to Noise Threshold	3
Apply Frequency Correction	False
**Charge Assigner: Voting v3**	
Bin Size (ppm)	1
Minimum Ions In Bin	1
Number of Charge Neighbors	2
Number of Isotope Neighbors	10

Table S2: Collision Cross Section center, area, and full width at half maximum (FWHM) for wtTTR conformers observed in Figure 2a (main text).

pH 3.4			
	**Center (Å^2)**	**Area (Å^2)**	**FWHM (Å^2)**
**Conformer 1**	1,706	120.51	127.26
**Conformer 2**	1,844	75.81	134.64
**Conformer 3**	2,029	148.58	139.55
**Conformer 4**	2,334	17.43	93.96
**pH 4.4**			
	**Center**	**Area**	**FWHM**
**Conformer 1**	1,687	120.35	113.00
**Conformer 2**	1,816	22.64	84.73
**Conformer 3**	1,939	30.32	101.10
**Conformer 4**	2,029	30.65	140.54
**pH 5.4**			
	**Center**	**Area**	**FWHM**
**Conformer 1**	1,689	111.11	107.11
**Conformer 2**	1,957	30.97	191.73

Table S3: Collision Cross Section center, area, and full width at half maximum (FWHM) for for L55P conformers observed in Figure 2b (main text).

pH 3.4			
	**Center (Å^2)**	**Area (Å^2)**	**FWHM (Å^2)**
**Conformer 1**	1,617	85.86	119.33
**Conformer 2**	1,741	65.41	155.02
**Conformer 3**	1,946	137.45	132.42
**Conformer 4**	2,123	21.00	166.75
**pH 4.4**			
	**Center**	**Area**	**FWHM**
**Conformer 1**	1,684	117.39	116.93
**Conformer 2**	1,821	67.33	167.72
**Conformer 3**	2,009	115.71	142.70
**Conformer 4**	2,139	28.86	106.30
**Conformer 5**	2,262	28.84	109.53
**pH 5.4**			
	**Center**	**Area**	**FWHM**
**Conformer 1**	1,704.53	142.95	153.91
**Conformer 2**	1,971.46	227.69	292.71
**Conformer 3**	2,376.15	26.67	188.04

Table S4: Masses of TTR oligomers from the DMT spectrum incubated at pH 3.4 in 100 mM AmAc at 4 °C for 24 h. (spectrum a) in main text Figure 1) .

Subunits of TTR	Measured Mass (Da)	Expected Mass (Da)	Difference (Da)
**1**	13,904.82	13,905.54	-0.72
**2**	27,808.04	27,811.08	-3.04
**3**	41,771.54	41,716.62	54.92
**4**	55,713.10	55,622.16	90.94
**5**	69,632.77	69,527.70	105.07
**6**	83,583.42	83,433.24	150.18
**7**	97,568.55	97,338.78	229.77
**8**	111,480.00	111,244.32	235.68
**9**	125,463.54	125,149.86	313.68
**10**	139,458.24	139,055.40	402.84
**11**	153,404.04	152,960.94	443.10
**12**	167,398.17	166,866.48	531.69
**13**	181,358.41	180,722.02	586.39
**14**	195,389.54	194,677.56	711.98
**15**	209,334.87	208,583.10	751.77
**16**	223,308.72	222,488.64	820.08
**17**	237,508.93	236,394.18	1,114.75

Table S5: Measured masses compared to theoretical mass of L55P oligomers. The measured spectrum is L55P incubated for 24 h in pH 4.4 100 mM AmAc at 21 °C.

Subunits of TTR	Measured Mass (Da)	Expected Mass (Da)	Difference (Da)
**1**	13,889.58	13,889.49	0.09
**2**	27,780.06	27,778.98	1.08
**3**	41,673.21	41,668.47	4.74
**4**	55,581.95	55,557.96	23.99
**5**	69,468.86	69,447.45	21.41
**6**	83,386.68	83,336.94	49.74
**7**	97,303.85	97,226.43	77.42
**8**	111,257.64	111,115.92	141.72
**9**	125,179.48	125,005.41	174.07
**10**	139,129.97	138,894.90	235.07
**11**	153,015.40	152,784.39	231.01
**12**	166,929.00	166,673.88	255.12
**13**	180,866.39	180,563.37	303.02
**14**	194,806.85	194,452.86	353.99
**15**	208,710.59	208,342.35	368.24
**16**	222,622.73	222,231.84	390.89
**17**	236,545.02	236,121.33	423.69
**18**	250,434.79	250,010.82	423.97
**19**	264,377.65	263,900.31	477.34
**20**	278,269.08	277,789.80	479.28
**21**	292,021.83	291,679.29	342.54
**22**	305,930.69	305,568.78	361.91
**23**	319,896.84	319,458.27	438.57
